# Cognitive offloading or cognitive overload? How AI alters the mental architecture of coping

**DOI:** 10.3389/fpsyg.2025.1699320

**Published:** 2025-11-21

**Authors:** Ginto Chirayath, K. Premamalini, Jeena Joseph

**Affiliations:** 1 Department of Social Work, Bishop Appasamy College of Arts and Science, Coimbatore, Tamil Nadu, India; 2 Department of Computer Applications, Marian College Kuttikkanam Autonomous, Kuttikkanam, Kerala, India

**Keywords:** artificial intelligence, cognitive offloading, cognitive overload, coping strategies, mental health, resilience

## Introduction

Artificial intelligence (AI) has moved from being a specialized technological tool to an intimate presence in everyday life. Smart assistants organize our schedules, predictive systems anticipate our needs, and therapeutic chatbots promise to listen when no human is available (Zhang and Wang, 2024). The diffusion of AI into mental health care is often framed in highly optimistic terms: technologies that reduce stigma, democratize access, and provide affordable, always-on support (Shankar Ganesh and Venkateswaramurthy, 2025; Sivasubramanian Balasubramanian, 2023). From meditation applications and emotion-tracking wearables to conversational agents offering cognitive-behavioral interventions, AI is marketed as an efficient and reliable partner in the pursuit of wellbeing (Balcombe, 2023).

Beneath this enthusiasm lies a paradox. On one hand, AI enables cognitive offloading: the use of external aids to reduce mental effort and conserve resources for more meaningful activities (Grinschgl and Neubauer, 2022; Risko and Gilbert, 2016). This process can support adaptive coping, helping individuals regulate stress and sustain mental health. On the other hand, the same tools may create cognitive overload: an erosion of introspection, over-reliance on algorithmic feedback, and anxiety induced by hyper-monitoring and optimization (Grinschgl et al., 2021; Skulmowski, 2023). The question is not whether AI is good or bad for mental health but how it is reshaping the very architecture of coping (Gerlich, 2025).

This article examines the psychology of this paradox. First, it places copings in mainstream psychological theory. Then it considers the promise of AI to lighten burdens through cognitive offloading. Next, it considers the risks of cognitive overload and theoretical insights that illuminate this conflict. Through practical illustrations, it demonstrates the double-sidedness of AI as copings partner on the one hand, yet destabilizer on the other. Finally, it identifies principles for design, clinical responses, and policy interventions required to ensure that AI complements, not undermines, human resilience.

## Coping in psychological science

Coping is central to psychological health. It encompasses the thoughts, behaviors, and emotional strategies individuals use to manage stressful events, regulate affect, and adapt to changing environments (Marroquín et al., 2017). Coping strategies have been categorized in multiple ways:

- Problem-focused coping, which seeks to address the stressor directly through actions such as planning or problem-solving (Gruber and Martic Biocina, 2024).
- Emotion-focused coping, which aims to regulate emotional responses through reappraisal, distraction, or relaxation (Oostvogels et al., 2018; Trudel-Fitzgerald et al., 2024).
- Avoidant coping, which minimizes distress through withdrawal or denial, sometimes maladaptive in the long term (Lin, 2022; Marr et al., 2022).

Coping is not a static trait but a dynamic process shaped by situational demands, personal resources, and social contexts (Depoorter et al., 2025). Historically, coping has always been supported by external aids. Religious rituals, cultural practices, and communal gatherings provided frameworks for resilience (Butler et al., 2025; Whitehead and Bergeman, 2020). Personal tools such as diaries, letters, or calendars extended introspection and memory, allowing individuals to regulate emotions and track personal growth.

AI represents a profound shift in this lineage. Unlike static tools or human communities, AI systems are adaptive, predictive, and personalized. They do not merely offer neutral support; they actively reshape the environment in which coping occurs (Shi, 2025). A diary records what one chooses to write; a mood-tracking app interprets and quantifies feelings, often presenting its interpretation as more authoritative than subjective experience (Brandsema, 2023; Yang and Zhao, 2024). This transformation raises new questions. How does the delegation of introspection to machines affect self-awareness? Does reliance on algorithmic recommendations weaken intrinsic coping mechanisms? And can AI be designed to scaffold rather than substitute the human capacity for resilience?

## The promise of cognitive offloading

Cognitive offloading is a well-established phenomenon in cognitive science. It refers to the delegation of cognitive tasks to external resources in order to reduce mental demand. People use notebooks, calculators, and smartphones to extend memory and problem-solving capacities. AI magnifies this process dramatically, providing not only storage but active analysis and prediction (León-Domínguez, 2024).

- AI in Mental Health Tracking: Mental health applications now integrate biometric sensors and self-reporting tools to track sleep, exercise, and mood. By aggregating data and presenting trends, these apps reduce the burden of self-monitoring (Bakker and Rickard, 2018). Instead of recalling fluctuations over weeks, users can visualize emotional patterns instantly. Such tools facilitate problem-focused coping, allowing individuals to identify triggers, monitor progress, and adjust behaviors accordingly (Blease and Torous, 2023; Lopes et al., 2024).
- Therapeutic Chatbots: AI-driven conversational agents, such as Woebot and Wysa, deliver micro-interventions based on cognitive-behavioral therapy. They can prompt reappraisal, encourage behavioral activation, and provide coping strategies in real time (Beatty et al., 2022). For individuals facing barriers to traditional therapy—such as cost, stigma, or geographic isolation—these tools lower thresholds to care. They exemplify emotion-focused coping, offering comfort and strategies at the moment they are needed (Coghlan et al., 2023; Inkster et al., 2018).
- Guided Reflection: AI-guided meditation programs and journaling assistants scaffold introspection. By generating prompts, suggesting breathing exercises, or helping articulate feelings, they encourage engagement with practices that might otherwise feel overwhelming. They reduce decision fatigue, making coping rituals easier to adopt and sustain (Park et al., 2024).
- Benefits for Coping: In these ways, AI promotes adaptive coping by:
  ○ Minimizing cognitive load associated with self-monitoring.
  ○ Reducing decision fatigue in stressful moments.
  ○ Providing immediate, context-sensitive interventions.
  ○ Enhancing self-awareness through data visualization.

Viewed positively, AI can function as a resilience amplifier. By offloading mundane or effortful processes, it frees mental resources for growth, creativity, and deeper social engagement.

## The perils of cognitive overload

The promise of offloading is shadowed by the risks of overload. When reliance on external aids discourages intrinsic engagement, coping may be weakened rather than strengthened.

- Erosion of Introspection: One risk is the erosion of introspection. Traditional coping often depends on reflective practices such as journaling, meditation, or conversation. When mood-tracking applications or predictive systems dictate interpretations of feelings, individuals may defer to the machine's account over their own experience (Brand et al., 2023). Complex emotions are reduced to numerical scores—“stress index: 75%”—flattening nuance and discouraging self-discovery.
- Outsourcing Resilience: A second risk is the outsourcing of resilience. If every moment of distress is met with algorithmic suggestions—“take three deep breaths,” “reframe your thought”—individuals may lose opportunities to cultivate independent strategies. Over time, this reliance may erode psychological immunity, leaving people ill-equipped to manage stress in contexts where technology is absent or unavailable (Gilboa and Nahum, 2022).
- Anxiety from Hyper-Monitoring: Finally, hyper-monitoring may itself generate anxiety. Wearable devices that continuously provide biometric feedback can create pressure to optimize every aspect of life. A poor “sleep score” may undermine subjective feelings of rest, while constant reminders to reduce stress can paradoxically heighten it. Instead of relieving burdens, AI may impose new demands that exacerbate distress (Inamdar et al., 2025).

These dynamics illustrate that AI is not a neutral partner but an active shaper of coping. Its interventions may scaffold resilience or foster dependence, depending on how they are designed and used.

## Theoretical perspectives

Several psychological frameworks help explain this paradox.

- Cognitive Load Theory: Cognitive Load Theory characterizes extraneous load (unnecessary effort), intrinsic load (embedded difficulty), and germane load (effort toward learning). Reducing extraneous load is beneficial, but technology that reduces germane load has the negative impact on deeper engagement. Extended to dealing with stress, AI will in the future alleviate present burdens but risk losing potential for long-term resilience building (Evans et al., 2024; Sultanova, 2025).
- Self-Determination Theory: Self-Determination Theory posits that autonomy, competence, and relatedness are basic psychological needs (Evans et al., 2024). AI may enhance competence by offering strategies but threaten autonomy by making choices on behalf of users. It may also compromise relatedness if machine interactions displace human relationships.
- Resilience Frameworks: Resilience research emphasizes both external supports and internal capacities. External aids are valuable but cannot substitute for the cultivation of intrinsic coping skills (Brockbank and Feldon, 2024). Overuse of AI may act as a crutch, preventing the strengthening of these internal resources.
- Social Cognition and Bias: Finally, research on social cognition shows that external interpretations shape self-perception. If individuals consistently rely on algorithmic interpretations of their emotions, they may internalize biased or limiting narratives (Le Cunff et al., 2025). For example, a system that over-detects anxiety may reinforce an anxious self-concept, even when variability exists.

## Integrating the theoretical frameworks: toward a unified model of AI—coping dynamics

Taken together, these four psychological frameworks form an interconnected system that explains how AI reshapes the mental architecture of coping. Cognitive Load Theory offers the foundational mechanism, describing how AI redistributes mental effort by externalizing cognitive tasks. This redistribution directly influences motivational dynamics described by Self-Determination Theory, as reductions in cognitive demand enhance coping only when individuals retain a sense of autonomy, competence, and relatedness. When these motivational needs are met, the process strengthens the internal capacities emphasized by Resilience Frameworks—promoting flexibility, persistence, and adaptive recovery over time. Conversely, if offloading undermines autonomy or fosters overreliance, resilience may deteriorate rather than grow. Overlaying these layers, Social Cognition and Bias Theory captures the reflective loop through which users internalize algorithmic interpretations of emotion and self, shaping how they perceive and evaluate their coping abilities. In this integrated view, AI's influence follows a coherent psychological sequence: cognitive redistribution affects motivation, motivation determines resilience outcomes, and feedback from algorithmic interpretation continually reshapes self-concept. This synthesis clarifies the theoretical thread connecting the frameworks and positions them not as parallel explanations but as interacting components of a unified model of AI-mediated coping.

## AI as a double-edged coping partner

The paradox of cognitive offloading vs. cognitive overload highlights the dual nature of AI as a partner in coping. Unlike static tools such as a notebook or calendar, AI is dynamic: it adapts, predicts, and responds in ways that make it both supportive and potentially disruptive (Grinschgl and Neubauer, 2022). Its role in mental health is therefore best conceived not in binary terms but as a continuum ranging from healthy offloading to risky overload.

At one end of this continuum lies healthy offloading. In this mode, AI technologies function as scaffolds—temporary supports that facilitate reflection, encourage adaptive strategies, and promote personal growth. For instance, an app that prompts users to pause and identify their emotions at stressful moments does not replace emotional regulation but nudges individuals toward engaging with their own cognitive and affective processes. Similarly, a chatbot that guides users through a breathing exercise can reduce acute anxiety while simultaneously teaching a transferable skill (Yang et al., 2025). In these cases, the technology acts like a training wheel, helping individuals stabilize until they are capable of maintaining balance on their own (Grinschgl and Neubauer, 2022).

At the opposite end of the continuum lies risky overload. Here, AI tools begin to dictate experiences rather than facilitate them. Instead of scaffolding reflection, they may prescribe emotions (“you are stressed”), deliver rigid coping instructions without space for adaptation, or condition users to rely on external prompts rather than developing internal regulation strategies. For example, if a mood-tracking device consistently tells a user that they are “anxious” based on biometric indicators, the individual may come to trust the algorithm's interpretation over their own felt experience. This risks undermining self-awareness and fostering dependence on the device (Gerlich, 2025). In such contexts, AI ceases to be a supportive partner and becomes a substitute for coping itself, replacing human agency with algorithmic authority.

The critical distinction, then, is whether AI operates as a scaffold or a substitute. Scaffolding is characterized by temporariness, adaptability, and empowerment: the goal is to strengthen internal capacities so that the technology becomes progressively less necessary (Britten-Neish, 2025). A meditation app that gradually reduces guided instructions, encouraging independent practice, exemplifies scaffolding. Substitution, by contrast, is characterized by permanence and dependency: the technology assumes responsibility for regulation in ways that diminish intrinsic skills. An AI system that requires continuous engagement for relief, without cultivating transferable strategies, exemplifies substitution (Diaz Alfaro et al., 2024).

Importantly, whether a given technology scaffolds or substitutes depends less on its technical sophistication than on its design philosophy, integration context, and patterns of use. Designers can encourage scaffolding by building features that promote reflection, variability, and user agency (Lahlou, 2025). Clinicians can integrate AI in ways that complement, rather than replace, therapeutic relationships. Users themselves can engage critically, treating AI as a tool rather than a determinant of their psychological states.

Understanding AI as a double-edged coping partner shifts the focus from whether AI is “good” or “bad” for mental health to how it is designed, deployed, and experienced. It invites researchers, clinicians, and policymakers to ask: Does this technology empower individuals to cope more effectively, or does it cope on their behalf? The answer to this question will determine whether AI becomes an ally in resilience or an architect of dependency.

## Real-world illustrations

The paradox is visible in multiple real-world contexts. Meditation applications such as Calm and Headspace provide structured routines that make mindfulness accessible (Huberty et al., 2020; Taylor et al., 2022). Yet dependence on guided sessions can leave individuals unable to practice independently, undermining self-sufficiency. Therapeutic chatbots offer another example. Evidence suggests that Woebot can reduce symptoms of depression and anxiety in young adults (Li et al., 2025). Yet prolonged use risks cultivating “pseudo-intimacy,” where trust is invested in a machine rather than fostered in authentic human relationships (Wasil et al., 2022). Wearable devices illustrate the risks of hyper-monitoring. Smartwatches that prompt stress reduction exercises can help prevent escalation. Yet constant biometric feedback may trigger obsessive self-monitoring, leading to heightened anxiety and detachment from subjective experience (Clarke and Draper, 2020). These cases show that AI can scaffold coping or create new vulnerabilities, depending on usage and context.

While the examples above illustrate how AI tools shape coping, their comparative analysis also reveals a systematic pattern across technologies. Meditation applications primarily reduce extraneous cognitive load, fostering short-term calm but risking dependence if self-regulation does not internalize. Therapeutic chatbots extend this mechanism into emotion-focused coping, enhancing accessibility and competence yet potentially weakening relatedness when human empathy is replaced by simulated interaction. Wearables, in turn, externalize physiological awareness, offering early warning and data-driven control but often amplifying anxiety through continuous self-surveillance. Viewed through the integrated theoretical model, these cases collectively demonstrate that AI-mediated coping outcomes depend on the balance between cognitive relief and motivational autonomy. When technology scaffolds reflection and supports agency, it strengthens resilience; when it substitutes intrinsic effort or distorts self-perception, it contributes to cognitive overload. Thus, the real-world evidence aligns with the theoretical continuum proposed in this paper, grounding the discussion in systematic, cross-case reasoning rather than isolated illustrations.

## Social and Ethical Implications

Besides individual psychology, there also are social and ethical effects of AI on coping. On the contrary, AI cuts down access barriers for mental health resources. Automated tools also reduce help-seeking stigmatization through anonymity. They gain access where there are few professional resources (Li et al., 2025). Conversely, there is the risk for self-surveillance culture normalizing with AI. Distress is individualized into data points rather than expressed through the community. Coping's social factor—help by family, friends, and cultural rituals—potentially is suppressed by the electronic individualized management

Algorithmic bias compounds inequities. Mental health AI trained on narrow datasets may misinterpret expressions of distress across cultures, genders, or age groups. For example, emotional expression varies widely across societies (Chen, 2025). If AI tools fail to recognize this diversity, they may deliver interventions that are ineffective or even harmful for marginalized populations. Finally, the privatization of coping risks weakening collective resilience. Communities have historically sustained resilience through shared rituals, storytelling, and mutual care (Jackson, 2020). AI interactions, by isolating coping within individualized digital exchanges, may erode these communal resources.

## Pathways forward

To reduce these risks, multi-level strategies are needed. Designers will need to favor scaffold over substitution. Features would include reflective prompts in place of instruction, delays with individual reflection opportunities, and disclosure on how recommendations are generated (Martinez-Martin, 2021). Diversity in the training datasets will need to be guaranteed to reduce the risk of bias. Clinicians will need to integrate AI as part of stepped-care models, where technology provides low-level support shifting to human intervention with increasing complexity (Hoose and Králiková, 2024). Mental health professionals training needs to concentrate on critical interaction with outputs instead of unconditional use. Policy protections will need to ensure the safeguarding of privacy, accountability, and inclusivity. Regulation will need to require transparent data protection procedures along with the testing of the systems for the AI for fairness and efficacy (Elendu et al., 2023). Finally, people need to learn how to use AI with intention. Treating technology as partner rather than replacement helps maintain agency. Setting aside “AI-free reflection time” can help maintain intrinsic introspection. Engaging in practices such as journaling, mindfulness, and relational conversation ensures that AI complements rather than replaces human coping (Balcombe, 2023).

## Conclusion

AI is reshaping not only how people live but also how they cope. The paradox of cognitive offloading vs. cognitive overload captures both the promise and the peril of this transformation. On one hand, AI can democratize access, reduce stigma, and scaffold resilience. On the other hand, it risks eroding introspection, diminishing autonomy, and fostering dependency.

The challenge for psychology is not to determine whether AI is beneficial or harmful in absolute terms. It is to ensure that AI empowers individuals to cope rather than coping on their behalf. This requires thoughtful design, careful clinical integration, robust policy safeguards, and intentional individual practices.

The future of mental health in the AI era will depend on maintaining this delicate balance. If technology can be designed and used to scaffold rather than substitute, it has the potential to become a genuine partner in wellbeing. If not, it risks becoming an architect of dependency. The task for researchers, practitioners, and policymakers is to ensure that AI strengthens, rather than weakens, the mental architecture of coping.
